# Collectanea Medica, Consisting of Anecdotes, Facts, Extracts, Illustrations, Queries, Suggestions, &c.

**Published:** 1815-10

**Authors:** 


### COLLECTANEA MEDICA,

CONSISTING OF

### ANECDOTES, FACTS, EXTRACTS, ILLUSTRATIONS, QUERIES, SUGGESTIONS, &c.

RELATING TO THE

*History or the Art of Medicine, and the Auxiliary Sciences.*

WE have expressed our intention of entering into the inquiry of Contagion with all the diligence which the importance of the subject demands. With this view, we began, in our last, to select such facts as may assist us in this undertaking. After we have accumulated as many as may seem necessary, we shall conclude by a review of Dr. Hosack's ingenious "Observations on the Laws governing the Communication of Contagious Diseases, and the Means of arresting their Progress," published at New York, and with a copy of which the author has honoured us.

All well-authenticated communications will be thankfully received, and inserted in such a manner as may arrest the attention of our readers. At present, we offer, from a contemporary Journal, as much of an ingenious paper relative to the last fever at Gibraltar, as relates to the present controversy.

"*Answers* to Queries relative to the Epidemic at Gibraltar, which were submitted to all the Medical Men in the Garrison by Mr. Frazer, now Deputy-Inspector of Hospitals.* By R. Amiel, Esq. Member of the Royal College of Surgeons in London, late Acting Surgeon to the Foreign Depôt, and Assistant-Surgeon to the Forces.

"Query, No. 1.—*When did you first observe the epidemic, and*

---

* Our readers will recollect another set of answers to these questions, which we selected from the Medico-Chirurgical Transactions. It is with much pleasure that we compliment Mr. Amiel on his superior perspicacity on certain points which we objected to as obscurely answered in the former paper.—Edit.

*do*

*do you attribute it to foreign introduction, endemic causes, or atmospheric vitiation? State facts in support of your opinion.*

"I observed the first case of the malignant fever which has lately prevailed in this garrison, when I visited the Lazaretto on the 19th August ultimo, having been absent, previous to that period, on account of the bad state of my health. A few cases of the same fever appeared afterwards in different parts of the town; and, by the 10th of September, they became so numerous as to leave no doubt about the existence of the epidemic. I had an opportunity more particularly to observe the origin and progress of that disease in the years 1810 and 1813; and, comparing the result of my observations with the history and cases of the malignant fever which raged here in 1804, I have no hesitation to give it as my opinion, that the epidemics which afflicted the garrison at those different periods are of the same nature, differing only as to the extent of their ravages, and sprang from the same source. I do not attribute it to foreign introduction, and I found my opinion on the following considerations: the rise and progress of our epidemics have never been traced, in a satisfactory manner, from a single point of contagion to a gradual number of individuals or families; and, instead of creeping slowly from one district to another, cases have appeared unconnected, and scattered at different points, and, in some instances, it has spread with the rapidity of the electric fluid, attacking persons who never had approached the sick, nor any assignable source of contagion.

An individual labouring under our epidemical fever, on being removed to a pure air and ventilated place, such as the Neutral Ground, or Europa Point, did not communicate the disease to those in the closest contact with him: this observation has been confirmed in many instances, during the epidemic of last year, amongst the foreign recruits quartered at the Brewery Barracks. The depôt consisted of between five and six hundred men, sixty of whom were permanently employed in the different departments in town, or as servants to officers. Those men, on being attacked by the epidemic (and I believe not one escaped it), generally came to the barracks, where they lay all night in a crouded ward, and, sometimes, by concealing themselves, they continued two days in the same place; yet I never observed, that either their breath or the effluvia of their bodies and clothes had proved infectious to their companions. Forty women of that depôt, who had been prohibited passing Europa Gate, remained perfectly healthy, although I had seen some of them sitting on the same bed where a man was lying in a fever. Out of the four thousand Spaniards removed last year to the Neutral Ground, a few died of the fever then prevailing in the garrison; but it is a well-known fact, that those only had the fever there who were already sick on going out, and that they did not communicate the disease to any of their neighbours or attendants. If this disease cannot be transplanted from this to such a small distance, could it have been transplanted thousands thousands of miles from America to Cadiz, and then from Cadiz to Gibraltar? and, if ventilation so effectually destroys the infectious quality of this fever in a place so contiguous to our atmosphere, is it to be believed, that it would have less efficacy on board a vessel, and especially in a boat, where a man or foul clothes must stay some time before they reach our shore?

The epidemics which have appeared at Gibraltar, as well as the epidemics which have raged at Cadiz, Carthagena, Malaga, Alicant, &c. &c. have always appeared about the latter end of the summer, or during the autumnal season. If the epidemic had the faculty of re-producing itself by contact with the sick, or with substances charged with the effluvia of the sick, would that faculty be inert during nine months of the year? and do other importable contagions consult seasons to make their ravages?

"The inefficacy of the various means which have been repeatedly employed to stop the progress of the epidemic, such as sending out the sick, shutting up their houses, prohibiting meetings of all kinds, &c. &c. and, on the contrary, the success which has evidently attended the measures of removing to a pure air those who appeared more susceptible of catching the fever, as was done last year with so many thousand inhabitants, and this year with the regiments who were growing sickly, clearly proves that our epidemical fever is not easily removed from its focus, nor easily exported to another place.

"Various cases of this fever have been observed in this garrison, while there prevailed no epidemic, nor any suspicion of an imported disorder; and I mention the two following as worthy of notice: the first has been witnessed by the Deputy Inspector of Hospitals in this place."

Two satisfactory cases follow of well-marked fever, with all the symptoms of the epidemic, which occurred whilst the garrison was healthy, and shew that the "epidemical fever has no need of the introduction of a foreign seed; but that it originates spontaneously here, and has, most probably, for its primary and essential cause, putrid exhalations floating in the atmosphere."

The author proceeds to show the places and manner in which that noxious air is generated to which he imputes the fever.

"The inhabitants being much limited for ground to build upon, have frequently placed the door and windows of the apartments on the same side; and have, by that means, made it impossible to have the air of the rooms sufficiently renewed.

"The ground for building being very dear, and house-rent excessively high, cellars and stables have been converted, in many places, into dwelling-houses to receive numerous families, without any regard to their health or accommodation; and, in other places, a great number of sheds have been constructed, in such a manner as to preclude access to ventilation, affording, besides, materials for putrefaction, by the decayed state in which they are frequently left.

"To overcome the excessive price of house-rent, the poor labouring bouring classes of people have been compelled to croud themselves in the same apartment, where it is not unusual to see three rows of beds one above the other, while some are lying on the floor, which is, in general, badly paved, and always damp.

"These people, going out early in the morning to procure their livelihood, are in the habit of shutting up the door and windows of the apartment, which are not re-opened until they come home in the evening, when they breathe an air deprived of its oxygen, or loaded with the noxious effluvia of their bed-clothes.

"It has been acknowledged by the Board of Health, in the proclamation dated August 27, that the first victims of the epidemic have been amongst these classes; and I am convinced, that such a deplorable state of misery has frequently given, among them, a malignant type to a disease which would otherwise have been a simple ephemeral fever; experience having shewn, that, whenever a fever appears in one of the individuals thus circumstanced, it attacks every one of them in succession, and always with increasing danger."

It appears afterwards that the sewers are ill constructed, and that putrid matter is permitted to stagnate, and that even the rain water, which, on account of the scarcity of that article, it is necessary to preserve, is often putrid. Many objections are judiciously answered, the most pointed of which are the long cessation of the epidemics, their re-appearance, and that frequently, when they have appeared, some imported sickness could be traced.

"If (says Mr. A.) the fever has not re-appeared from 1805 to 1810, and more lately, in 1811 to 1812, it must be ascribed to that unsteadiness and unaccountable contingency which mark other diseases in their virulence and re-appearance. And lastly, the arrival in the garrison of one or more persons, ill of this malignant fever, at the first breaking out of the epidemic, does not prove its foreign origin, or its propagation by contagion; for, on the one hand, this fever has broken out in places where there was not the least possibility of foreign introduction; and, on the other, a number of people labouring under it have sometimes been landed in other places, without injury to the health of the inhabitants. In support of this fact, I submit the following memoranda:

"'At the commencement of January, 1812, about 460 foreign recruits arrived in this bay from the Spanish coast: part of them were landed in this garrison on the 13th of that month, but they were re-embarked the next day on board the Downs and Langley Transports, to proceed immediately to England. By some accident, the Downs lost the convoy in the night, and came back to this bay, where she lay on the 28th, when I was desired to go on board, as some of the recruits had been taken ill. On my first visit, I found five or six men with a fever, one of them very ill; and I observed, that, besides their being in a crowded state, they were in great want of clothes; a circumstance which induced them constantly to stay between decks, in order to avoid the inclemency of of the weather. On the 30th of January, the sick, whose number had increased to thirteen, were landed and brought to the hospital of foreign recruits; and, on the 2d of February, eighteen more of the same men, who had been taken ill, were likewise landed and sent to the hospital of the 7th R. V. B. in charge of Dr. Lamert: the number of the sick, however, continuing to increase among them, an hospital was established afloat, on board the transport Edward, and I believe, no less than forty of the same men were admitted into it in the course of a very few days. The fever in all those men presented a type more or less continued, was attended with irritability of the stomach, bilious vomiting, yellowness of the skin, &c. &c.; and had, in every symptom, a perfect resemblance to the epidemic which has prevailed in this garrison.'

"The circumstances which preceded the breaking out of this fever, exclude every suspicion of its having been excited by the introduction of a foreign poison; and its not having spread to the people lying in the same wards with them ashore, proves that it was not a disease propagated by a specific contagion, but a disease generated in that filthy, crowded, and unventilated vessel—which may serve to establish the fact, that the malignant fever of our epidemic may originate, in this warm latitude, in any place where the same circumstances are to be found."

We pass over the 2d, 3d, 4th, 5th, 6th, 7th, as not immediately to the purpose, excepting the following history of a family, which, though introduced with a different intention, shews in a most pointed manner that *places* are contagious more than the *sick themselves*.

"On the 20th October, I was called to see Jacinto Reys, a carpenter in the dock yard, attacked with fever; he had been working three or four days on board the *San Juan*,* where he was taken ill. The leading symptoms were, severe head-ache; eyes inflamed; dorsal pains; pulse very quick and full; great irritability of the stomach; constant retching; and vomiting of a bilious-like matter, &c. &c. The disease went through its periods to the 7th day, when a free perspiration brought on a favourable crisis. On the 22d of October, Andrew Reys, an elder brother of the patient, who did not live in the same house, but who had visited him the day before, and had been sitting for some time on his bed, was taken ill with the same leading symptoms; and in the course of three days, the mother, the grandmother, a child, and a person who lived on the same floor, were taken ill of a fever presenting the same symptoms as Jacinto Reys. Andrew Reys died of that fever on the 26th of October, and the rest of the family were sent to perform quarantine on the neutral ground, where the grandmother died.

"No new cases appearing in the garrison, the quarantine was

---

* "See page 138, Burnett's Mediterranean Fever, and Edinburgh Journal for October, 1812.

taken off on the 24th of November, and they were allowed to return to their homes. A younger brother, however, named Joseph Reys, 14 years old, who had been very healthy during the time of the quarantine, was, three days after returning to his house, seized with a fever which presented the same symptoms as his brothers', but in a more aggravated form; on the 3d day, a vomiting of black matter came on, and he died on the 4th day from the attack. The patient was visited by the greater number of medical officers in the garrison; the family was again sent to the neutral ground, and their furniture burned.

"On examining with attention the circumstances of the cases which occurred during the fevers prevailing in this garrison in that year, it appears to me evident, from the facts which directly came under my knowledge, that atmospheric causes, more or less powerful, according to the different changes of seasons, gradually directed the form of those fevers, from the mild bilious remittent in July and August, to a more aggravated one in September and October, attended with great irritability of the stomach, but with remissions still distinct; and lastly, in November, to that malignant type which brought on dissolution in four days, preceded by black vomiting. The exciting causes of the fever, which attacked the whole of the unfortunate family of Reys, must have had the same origin; but those exciting causes were more concentrated, and had acquired greater energy when they attacked Joseph Reys, its last victim."

The 8th question relates to the *possibility of the same person being twice affected.*—Such instances, it is answered, are very rare, but have occurred.

The 9th question enquires, *whether any causes influenced the progress and decline of the fever.*—The answer refers the whole to the condition of the atmosphere.

The 10th question is, *Was it contagious or infectious? The reasons for supposing so or not.* How much we regret that the precise definition of these two words, in the opinion of the querists, did not attend the queries. For want of this, we cannot wonder if the answer is less perspicuous than we have had occasion to remark and to applaud on every other occasion.

"Our epidemic being produced by local causes, not reaching the people beyond this side of the Rock, cannot correctly be deemed contagious or infectious, but merely a simple, primary, and idiopathical fever, taking its type from the energy and concentration of those deleterious causes which, floating in our atmosphere, uniformly influence a great number of persons, and, under certain circumstances, induce in them a uniform and general aberration from the healthy state, thus constituting an epidemical disorder. This, I believe, is merely the character in which the fever has appeared here during the present and preceding years, at the commencement of the season. The primary causes, however, continuing to operate, the putrid effluvia, arising from febrile bodies under an insalubrious atmosphere, have acquired, in some instances, stances, a contagious or infectious quality; that is to say, the faculty of producing symptoms resembling those which constituted the disease from whence they originated, and of propagating that disease from one body to another, by the laws of *contagion*, which are equally terrible and mysterious.

"The transition or aggravation from a simple epidemical fever to a contagious one, has been remarked by many writers. We read, in Dr. Clark's Collection, page 61: 'The remittent fever, contracted at unhealthy harbours, where ships touch for refreshment, from inattention to cleanliness and ventilation, acquires a high degree of virulence and *contagion*.' Dr. Lind on Hot Climates, p. 138: 'In the year 1741, no sooner had the rainy season set in at Carthagena, where the English troops lay encamped, than the remittent fever, then remarkably malignant, became also *contagious*, and destroyed the greatest part of the army.' Guyton Morveau, in his Traité de moyens de désinfecter l'Air, page 244, says, 'La plus terrible des contagions, et en méme temps la plus commune, est celle que produit le grand nombre des personnes affectées de la même maladie, quels qu'en soient le caractère et l'origine.' And Mr Assalini, in his work, entitled, Observations sur la Maladie appellé Peste, page 11: 'Some epidemic diseases become *contagious* solely from a number of persons being crowded together in one place, and especially in ill-ventilated hospitals.'

"Relying on such respectable authorities, I am strongly inclined to think, that the endemical fever of this climate, appearing now and then under the type of bilious remittent, has attacked in the same manner a multitude of persons exposed to the same atmospheric causes, and has constituted our epidemical disease; but that disease, epidemical only in the abodes of filth and confined air, has acquired, in some circumstances, an infectious property, which, together with predisposing causes, such as fatigue, intemperance, heat, &c. and above all, the painful apprehension of soon becoming a victim to a scourge which every day cut off so many people—an apprehension which staggers the most resolute—has increased the malignity of the fever, and rendered it pernicious to the highest degree. The infectious quality, however, being only accidental, has been lost whenever the patient, or his foul clothes, have been transferred to a pure air and ventilated place, as has been confirmed by experience with the people removed to the neutral ground, and with the foreign recruits quartered last year at the Brewery Barracks.

"My opinion relative to the epidemical fever having, in some instances, acquired an infectious property, is derived from the history of that fever in the Rey's family, which I mentioned before; the circumstances of Andrew Rey's not living in the same place, not having been exposed to other remote causes to which the rest of the family might have been subject, form a strong presumption that he caught the fever by sitting so long on the bed of his feverish brother. My opinion is derived likewise from my own experience in the hospital of foreign recruits, where three men and myself

 were were taken ill with a fever in consequence of our attending thirty-four men who landed from Alicant, as stated in the preceding pages; and lastly, from the statements of some patients, who have been able to trace the moment and the track of the infection to their own persons, in the manner which has been so well described by Dr. Trotter, in his Medicina Nautica, pages 213 and 214."

The remaining questions lead, by their answers, to answers connected with the subject of contagion.

"No. 11.—*Were other acute diseases observable during the epidemic season?*

"I have had under my care different acute diseases, such as catarrhs, pneumonia, dysenteries, &c. &c. during the prevalence of this and of the preceding epidemics.

"No. 12.—*Were the symptoms the same at the commencement of the epidemic, as towards the end of the season, particularly in fatal cases?*

"I believe the symptoms of the epidemic have been the same from the commencement to the end of the season; but, as far as I have been able to ascertain, they have grown milder since the weather has grown cooler, and I have not heard of late of any cases attended with that intense malignity and rapid dissolution, so frequent in September and October.

"No. 13.—*Have you observed the disease particularly arise in any class of individuals in the habit of constant communication with each other? or have you observed it to have been particularly severe in certain tents, guard-rooms, or barracks? if so, report upon them.*

"I believe it particularly arises in that class who are exposed to the sun or great fatigue, are reduced to low living, or indulge in drunken excesses. It is particularly severe among those who inhabit ill-ventilated places, and are in the habit of crowding themselves in the same apartment, as is frequently the case with the labouring people of this garrison. When I had charge of the 26th regiment last year, I observed that the soldiers of that regiment, who had been on Rosia guard, were more subject to be taken ill than those who had been in the other guard rooms.

"No. 14.—*At what period, after the exposure to exciting causes, did the disease generally manifest itself?*

"I cannot give a positive opinion on this query, as I want experience to ascertain if nature pursues in this a regular course; but from the circumstance of people having been taken ill on the first day of their landing here, and from the information I had, that people who fled from this garrison in 1804, were taken ill on the coast of Portugal many days after they had left this place, I am induced to believe that the disease may be put into action at any time from the first moment of exposure to a period of fifteen or twenty days.*

* "Vide Johnson's Essays on Tropical Climates, p. 78, where he computes a medium of twelve or fourteen days."

"Such

"Such are the answers which my observations and experience enable me to give to the proposed queries. During four years and a half that I have been in this garrison, I have constantly had the charge of a depôt, into which have been gradually admitted near twelve thousand deserters or prisoners from the French army. The wretchedness and hardships those men had undergone on the Spanish coast, predisposed them strongly to diseases, and the opportunity of witnessing among them the types which the fever assumes in this climate has been unfortunately too frequent; and from observations carefully repeated I derive my opinion, that the bilious remittent of the summer, and the epidemic of the autumn, are produced by the same causes more or less concentrated, and acting more or less generally; and that, although the autumnal fever might have been infectious in some instances, its infectious quality may be easily prevented; and it is to the destroying of its primary sources that our wishes and labours must be earnestly directed.

"Among other means which government, in its wisdom, may think proper to adopt, in order to obtain this desirable end, there is one which has often occurred to my mind during my professional intercourse with the sick poor of this garrison, and which I take the liberty of submitting to his Excellency the Lieutenant-Governor's paternal solicitude; namely, the establishment of an hospital, into which should be admitted any poor person labouring under a fever or any acute disorder. Such an establishment, which appears the more necessary as many of the poor of this place are only here *en passant*, would combine the double advantage of stifling the germs of any infectious fever, by preventing its spreading to other individuals, and of bringing the patient under that medical assistance which he cannot get at his own house on account of his poverty, and which he avoids to claim for fear of being removed to the Lazaretto, which has lately become an object of great terror to the poorer classes in the fortress."

---

We promised in a former Number to give those parts in Dr. Spurzheim's new edition, which may be truly called additions. The principal of these are his remarks on Sir Everard Home.

"Dr. Baillie, in the fourth edition of his excellent work on Morbid Anatomy of the Human Body, London, 1812, p. 448, in a note, says, 'A distinguished author has, in a late publication, insisted very strongly upon the existence of an immediate communication between the two lateral ventricles of the brain, and has expressed great surprize that it has been denied by several teachers of anatomy in London. Without entering into any dispute about this matter, which in itself is of no importance, I shall briefly mention what appears to me to be the real state of the circumstances. The fornix, at its anterior extremity, lies loose upon a part of the thalami nervorum opticorum, and there is a small chink on each side of the fornix leading obliquely downwards from the lateral ventricles to the anterior extremity of the third ventricle. While the the fornix is allowed to remain in its natural situation, there seems to me to be no immediate communication between the lateral ventricles. But, when the fornix is elevated, which may be very easily done, then the lateral ventricles communicate directly with each other, and the communication is more or less according to the degree of the elevation. It may be said, that the lateral ventricles still communicate together by means of the third ventricle. This, however, is not properly an immediate communication between the two lateral ventricles, unless any two cavities, which communicate with a third, may be properly said to communicate directly or immediately with each other.'

"As Dr. Baillie is disinclined to dispute about this matter, he might, without injury to his statement, have omitted the reasoning into which he enters at the end of his note, where he says that two cavities communicating with a third cannot be properly said to communicate directly or immediately with each other. The essential point to be considered is the communication of the lateral ventricles with each other. I shall, however, state how far we agree with Dr. Baillie, and in what respect I consider his statement as incorrect.

"In the first volume of our large work on Anatomy and Physiology of the brain, published at Paris, 1810, we have mentioned that the lateral ventricles are in communication, as soon as the fornix is elevated. For this reason, we have stated, that a small quantity of fluid may indeed be accumulated on one side; but that the opinion of certain authors, according to which there are large hydrocephali on one side, seems to us without foundation in observation, and incompatible with the structure of the brain.

"Dr. Baillie says that he mentions what appears to him. I well know, that in many things we must be satisfied with appearance; but on this point of anatomy I speak in a positive way, because the real structure can be shewn in every head of man and animals. The fornix, *in its whole extent*, is separated from the pretended optic thalami. Dr. Baillie then is, with many other anatomists, mistaken in stating, 'that the fornix at its anterior extremity lies loose upon a part of the thalami nervorum opticorum, and that there is a small chink on each side of the fornix leading obliquely downwards from the lateral ventricles to the anterior extremity of the third ventricle.' This opinion is also in contradiction with the other assertion that 'the fornix is easily elevated.' This, indeed, would be impossible without a separation between the fornix and thalami.

"The fornix does not lie loose merely at its anterior extremity: its separation is much more considerable, and may be easily demonstrated as we have represented it in our large work. There, a great part of this separation is visible in Plate XIV. and XVII. between γ, 59, 60, and 73; and its continuation is shewn Plate XV. from 73 to 72 and 70.

"The general statement in the text also, that, 'when the quantity of water is very considerable, the fornix is raised, at its anterior rior extremity, in consequence of its accumulation,' is incorrect. This is the case only when the water is collected in the anterior cornua: but, when the accumulation of water begins in the lateral and posterior lobes, the posterior part of the fornix is sooner elevated than the anterior part.

"There is still another observation of Dr. Baillie to which I cannot agree. He says, that this matter in itself is of no great importance. I think knowledge and truth are always more important than ignorance and error; and in anatomy the structure of any part cannot be indifferent. I prefer, for instance, to *know* that between the fornix throughout its whole extent, and the pretended optic thalami, for above three inches, there is a perfect separation; rather than to be satisfied with the *appearance* that only a small chink separates the fornix at its anterior extremity from the optic thalamus.

"In order to shew that it is really important to be acquainted with the structure of this part of the brain; that it is worth while to determine it; and that it is not sufficient to state what it appears to be, I shall quote a fact, as it has been lately published in the Philosophical Transactions for the year 1814, Part II. p. 473, read to the Royal Society by Sir Everard Home.

"In a boy the enlargement of the head was perceived at three months, and increased for three years, and then appeared to be stationary, and the child till that period was sensible. The upper part of the skull from that time began to ossify, and in three years more there was only an irregular space of the os frontis remaining open. The child continued sensible till three years old, and then became gradually less so, did not know what he did, heard sounds, but could not see. At six years old he died. The child was three feet three inches high: the skull twenty-seven inches round: the water, contained in the two lateral and third ventricles, was six ale pints and a half in quantity. The cerebrum formed a thin case of medullary substance, surrounding this cavity. The cerebellum was entire.' In a note, Sir Everard Home adds, 'The lining of the lateral ventricles was tough: the septum lucidum elongated, so that the corpus callosum was raised up close to the skull, the falx of the dura mater being nearly obliterated. The water in the third ventricle had split the fornix and septum lucidum into two, and the thin membranes of the septum had holes in them, making a communication between the third and lateral ventricles. The substance of the brain surrounding those cavities, as well as the pia mater covering it, had no convolutions; there was a continued smooth surface. On the right side, upon which the child was usually laid, there were no remains of medullary or cortical substance; and there the pia mater and dura mater adhered together. There was no remaining brain between the third ventricle and sella turcica. On the left side of the left hemisphere, the medullary and cortical substance was only half an inch thick. The corpora striata and thalami nervorum opticorum were small and tough; the union between the thalami was elongated into a broad flat ligament. The two two commissures and iter ad infundibulum had the natural appearance. The olfactory nerves were tough and small; the optic nerves had no medullary pulp; the other nerves going out of the skull had undergone no change.'

"I should, indeed, never have believed that such statements could be made, unless I had actually seen them in a printed work. I do declare, that *such things* as are here stated could only *appear* so: they are in absolute contradiction to nature and to reason. To him who really knows any thing of the structure of the brain, it would be quite sufficient to quote them. As, however, I write also for other readers, who may be desirous to become acquainted with the structure of that organ, I shall establish my assertion. First then, anatomically speaking, it is quite impossible that the two commissures and the iter ad infundibulum should have had the natural appearance, and that, at the same time, there should be no remaining brain between the third ventricle and sella turcica. Those who have our large work may consult Plate XI. and those who possess the opportunity may examine nature itself, in order to be convinced how erroneous such statements are. Plate XI. 61, is the anterior commissure; 44 the posterior; and 22 the infundibulum: this latter part is situated immediately on the sella turcica. In this case, moreover, the cerebellum was entire. Now, considering that the space between 22, 61, and 44, as well as the whole cerebellum, was entire; and the statement, that at the same time there was no brain between the sella turcica (the bony mass below 22 and 32) and the third ventricle (M M), it is evident that those alone will believe this who place credit in assertions in the direct ratio of their impossibility.

"'On the right side, upon which the child was usually laid, there were no remains of medullary or cortical substance, and there the pia mater and dura mater adhered together.' I will not ask, how Sir Everard Home accounts for the want of cerebral substance on the right side, while there yet existed the pia mater, which, however, is composed of the blood-vessels of the brain, &c.—The corpus callosum, the fornix and the two commissures, however, could not possibly exist without brain on the right side, because at least one-half of these parts originates from the lateral mass of the brain.

"'The water in the third ventricle had split the fornix and septum lucidum into two, and the thin membranes of the septum had holes in them, making a communication between the third and lateral ventricles.' This is also quite impossible. The fornix, in the middle line, is adherent to the corpus callosum, and not to the pretended optic thalami between which the third ventricle is formed. Hence, if water be collected in the third ventricle, and elevate the corpus callosum, the fornix must follow it; and, if the fornix was really split, I declare that this circumstance must have been the effect of a violent mode of procedure. Of this I am well convinced from my own observation; and I confidently refer the point to the future statements of accurate anatomists. At all events,

 this this paragraph shews, that practitioners in general do not yet sufficiently know, how, in hydrocephalic heads, the communication between the lateral ventricles is established.

"The reasoning on this observation by Sir Everard Home is still less satisfactory. A summary of the case may be sufficient. 'Six pints and a half of water were contained in the two lateral and third ventricles; the *cerebrum* formed a thin case of medullary substance surrounding this cavity; the substance of the *brain surrounding* those cavities, as well as the pia mater covering it, had no convolutions; there was a *continued* smooth surface; the lining of the lateral ventricles was tough;—and——on the right side there were *no* remains of *medullary* or *cortical substance!* and on the left side they were half an inch thick.' Are the existence of lateral ventricles, a thin case of brain, brain half an inch thick, and no brain at all, synonimous terms, since they are here employed to designate the same general fact? !!!

"Far be it from me to doubt the general accuracy of observations which characterizes the statements recorded in the transactions of a society which the immortal names of so many accurate observers and profound philosophers have rendered for ever illustrious. As accuracy and precision are the essential basis of any observer, I will *not* believe that such is the general inaccuracy even of Sir Everard Home's observations: I suppose that it is only in this particular case that Sir Everard Home can possibly have committed any oversight of the kind, since, I have no doubt, he has paid no *great* attention to the brain and nervous system. And although the statement which he has here made does involve a few contradictions, yet the thing *appeared* so to him."

"Sir Everard Home, in his Observations on the Functions of the Brain,* seems to maintain that there is a certain quantity of water in all brains. He even says 'Facts appear to point out the use of the water in the ventricles in the brain, and they account for the great variety which is met with in the form and extent of the posterior cornua, of the lateral ventricles, their size varying according to the quantity of water which is necessary to keep up the pressure required.' He says, 'pressure to a certain degree, uniformly kept up, is necessary for the performance of the healthy functions of the cerebrum, and any increase or diminution of this pressure puts a stop to them.' This reasoning is founded on the observation, that often large hydrocephalic heads continue to perform the functions of the brain.

"It is certainly true, that the cavities of the brain vary according to the quantity of collected water. They are, however, very different in the natural state, when no water is contained in them. It is, however, quite incontestable that water is either the effect of disease, or is collected after death; for in animals which are killed, or in men who die suddenly by a violent external cause, no water is ever found in the ventricles.

* "Loc. Cit. p. 471."

"Physicians, however, are not agreed where the water is contained in hydrocephalic persons. I speak here only of those whose skull is distended beyond the natural size; for there are two other varieties of this disease, which are very important in the practice of medicine, but which do not belong to this subject. Dr. Baillie, in his Morbid Anatomy, in treating of the symptoms of hydrocephalus, has entirely neglected this difference. Sir Everard Home, in his Observations on the Functions of the Brain,* also confounds the chronic and acute hydrocephalus. He thinks that 'the quantity of water may be much increased without material injury to the functions of the brain, when the skull is not ossified; but, after that period, even a few ounces in the lateral ventricles have been known to produce as much undue pressure, as to bring on head-ache, general uneasiness, a sensation as if the head were too large, loss of spirits, convulsions, loss of memory of recent events, idiotism, insensibility and death.' Now all the cases which he here states are of an acute nature; and ought to be distinguished from those of the chronic hydrocephalus. The acute dropsy of the brain is an effect of another disease: but the chronic hydrocephalus constitutes a peculiar morbid state."

"The explanation of these phenomena is easy to those who know the structure of the convolutions of the brain. From this it follows that in large hydrocephalic persons the brain is not disorganized, but that the cerebral fibres have only been changed from their vertical into a horizontal direction. Now the functions of the intellectual faculties do not depend essentially on the vertical, horizontal, or inclined position of the cerebral fibres; and their manifestations may continue without great derangement, if the pressure of the water upon the brain be not too strong, but only act upon it by degrees. It is also possible, that the cerebral fibres may be elongated without the internal organization being destroyed. When an excrescence pushes the eye-ball out of the orbit, the optic nerves are sometimes elongated without losing the faculty of sight. Thus all the arguments which have been founded on hydrocephalus, in order to prove that the brain is not exclusively the organ of the soul, fall to the ground.

"It is sufficiently well known that we were the first who explained how the structure of the brain admits so wonderful a change in its form, without being disorganized. Yet there are writers who speak of these facts as perfectly known to them for a long time past. Sir Everard Home after having mentioned the history of a boy whose hydrocephalic head measured thirty-three inches in circumference, and whose faculties were unimpaired, says: 'The preceding facts explain satisfactorily that the cerebrum is made up of thin convolutions of medullary and cortical substance surrounding the two lateral ventricles, which are unfolded when the cavities of those ventricles are enlarged, and in this unfolded state the func-

* "Phil. Trans. Part. II. 1814, p. 474."

tions belonging to this part of the organ can be carried on.' Now, our Memoir announcing this truth was presented to the National Institute of France in March, 1808; and was, by their report in the same year, made known all over Europe.—Mr. Home's paper respecting it was read to the Royal Society in May, 1814, namely, six years after our discovery was thus laid before every learned society in Europe! Before Sir Everard Home read his paper, I had made the demonstration of the structure of the brain in the Medico-Chirurgical Society in London. Will he maintain that he never heard our discovery spoken of, not even in so vague a manner as he has related it?"

"It is impossible to determine the functions of the cerebral parts by their mutilation.

"Sir Everard Home,* in his Observations on the Functions of the Brain, read at the Royal Society on the 26th May, 1814, seems to trust to a peculiar means of determining the functions of the cerebral parts. He says 'the various attempts, which have been made to procure accurate information respecting the functions that belong to individual portions of the human brain, having been attended with very little success, it has occurred to me, that, were anatomical surgeons to collect in one view all the appearances they had met with, in cases of injury to that organ, and the effects that such injuries produced upon its functions, a body of evidence might be formed, that would materially advance this highly important investigation.' He then informs us, that he has brought together certain observations, 'stating them as so many experiments upon the brain, with the conclusions which tend to elucidate this particular inquiry.'

"Let us first hear his observations. We read† 'that in the torpid state, commonly attendant upon any violent shake being given to the brain, the senses are so much impaired, that little information can be gained respecting the effects produced upon the internal organs;—that a *coup de soleil* is sometimes accompanied by delirium, loss of speech, and the power of swallowing:—that blood extravasated in the lateral and third ventricles was attended by repeated fits of vomiting and coma:—that coagulable lymph spread over the union of the optic nerves, the pineal gland and tuberculum annulare, was followed by permanent contraction of the muscles between the occiput and vertebræ of the neck, dilatation of the pupils, and a great degree of deafness;—that the formation of pus under the dura mater covering the right hemisphere was accompanied by delirium succeeded by coma;—that a tumor in the substance of the posterior lobe of the brain was attended with derangement of the functions of the stomach and bowels, and with double vision;—and that a deep wound into the right anterior lobe of the brain, attended with inflammation and suppuration, produced no

*" Phil. Trans. for the year 1814, Part II. p. 469."

† " Sect. II. p. 477, &c."

sensation sensation whatever, the senses remaining entire, and the person not knowing that the head was injured. In a case, also, in which the tuberculum annulare had become so hard as with difficulty to be cut with a knife, a considerable quantity of earthy particles having been intermixed with the medullary substance of the crura and other parts of the cerebellum, and the cerebrum and upper part of the cerebellum being unusually soft, the effects were, that the boy had been an idiot from birth, never walked, spoke and understood what was said, often went three days without food, and so on.'

"I suppose Sir Everard Home did not intend to state such facts as quite new and unobserved; for every one who is but half acquainted with the history of the healthy and diseased state of the brain knows, that many authors have related similar facts. We learn, however, from their writings that similar affections of the brain have often produced no perceptible derangement in the mind. I only maintain, that these means are quite unfit to point out the functions of the brain, and that any hope of it from such a source is in vain; and I may support my opinion by the observations of Sir Everard Home himself. He accordingly speaks of a body of evidence which might be formed, and of conclusions which tend to elucidate this particular inquiry, but he has not drawn even one inference. In the various pathological affections of the brain, he has observed head-ach, giddiness, faintness, loss of memory, want of sleep, delirium, mania, depression of spirits, melancholy, apoplexy, idiotism, hissing noise in the ears, deafness, blindness, loss of speech, irregular pulse, stupor, the mouth drawn to one side, numbness of the arms and legs, spasms in the lower extremities, stumbling in walking, pain between the shoulders, nausea, retching, slow action of purgative medicines, vomiting, convulsions, &c. Is Sir Everard Home then inclined to draw the inference that the brain is the organ of these symptoms, or of the states which are opposite to them. This is, I think, sufficient to shew an intelligent reader that, in this way, we never shall be able to determine the peculiar functions of the cerebral parts."

"The skulls of many madmen are also thicker than usual, and, even when they are not thicker, they are at least dense and heavy. This phenomenon is probably an effect of some alteration in the brain, and it furnishes a new proof of the influence of the brain upon the skull.

"Sir Everard Home, in his observations on this object, errs in classing together depression of the skull by external violence and thickening of the different portions of the skull. He has no idea of the diminution of the brain as the cause of the increased thickness of the skull; but always considers the thickening of the skull as the cause of the changes which he observed in the brain. Dr. Baillie, in his Morbid Anatomy, does the same. Often, however, the disease begins in the brain, and is propagated to the skull. Still, I do not deny that the skull may be diseased, and exercise a morbid influence upon the brain. I have, indeed, stated this in speaking of the pretendedly ossified brains."

These are the principal additions in the new edition; the other alterations are chiefly confined to arrangement. CRITICAL

